# Stable room-temperature ferromagnetic phase at the FeRh(100) surface

**DOI:** 10.1038/srep22383

**Published:** 2016-03-03

**Authors:** Federico Pressacco, Vojtěch Uhlίř, Matteo Gatti, Azzedine Bendounan, Eric E. Fullerton, Fausto Sirotti

**Affiliations:** 1 Synchrotron-SOLEIL, Saint-Aubin, BP 48, F-91192 Gif sur Yvette Cedex, France; 2 Center for Memory and Recording Research, University of California, San Diego, 9500 Gilman Drive, La Jolla, California 92093-0401, USA; 3 Laboratoire des Solides Irradiés, École Polytechnique, CNRS, CEA-DSM-IRAMIS, Université Paris-Saclay, F-91128 Palaiseau, France; 4 European Theoretical Spectroscopy Facility (ETSF), F-91128 Palaiseau, France

## Abstract

Interfaces and low dimensionality are sources of strong modifications of electronic, structural, and magnetic properties of materials. FeRh alloys are an excellent example because of the first-order phase transition taking place at ~400 K from an antiferromagnetic phase at room temperature to a high temperature ferromagnetic one. It is accompanied by a resistance change and volume expansion of about 1%. We have investigated the electronic and magnetic properties of FeRh(100) epitaxially grown on MgO by combining spectroscopies characterized by different probing depths, namely X-ray magnetic circular dichroism and photoelectron spectroscopy. We find that the symmetry breaking induced at the Rh-terminated surface stabilizes a surface ferromagnetic layer involving five planes of Fe and Rh atoms in the nominally antiferromagnetic phase at room temperature. First-principles calculations provide a microscopic description of the structural relaxation and the electron spin-density distribution that support the experimental findings.

Electronic and magnetic properties are strongly modified by symmetry breaking and reduced dimensionality at surfaces and interfaces. Spatial confinement and interface engineering can lead to fundamental discoveries of new phases and functionalities (e.g. topological insulators^1^ or interface phenomena in complex oxides^2^,^3^) revealing emerging behavior that is not present or is very different in the bulk^4^,^5^. In this regard the FeRh compound is a promising material showing a metamagnetic first-order phase transition above room temperature that is of great interest for future technologies such as heat assisted magnetic random access memories (HA-MRAM)^6^,^7^, magnetic cooling^8^,^9^ and spintronics devices^10^,^11^,^12^. The transition is a change of the magnetic ordering of the Fe moments from antiferromagnetic (AFM) at room temperature to ferromagnetic (FM) above 380 K, which is followed by the appearance of a net magnetic moment on the Rh atoms^13^,^14^,^15^. This metamagnetic transition is commensurate with an isotropic expansion of the lattice structure^16^,^17^ and a sizeable variation of the magnetoresistance^18^,^19^. The complexity of the phenomenon therefore raises fundamental questions about the interplay between magnetic order, electronic properties and atomic structure. This has triggered a number of studies and experiments over the last decades with the aim to explore the nature and driving mechanisms of this magneto-structural transition^20^,^21^,^22^,^23^,^24^,^25^,^26^.

While most of the studies were focused on the behavior of bulk samples, in recent years particular interest has grown around the properties of FeRh in presence of interfaces with substrates^27^ and overlayers^28^,^29^,^30^,^31^,^32^, also with the aim to identify their influence on the nucleation of the FM domains associated with the phase transition^23^,^32^,^33^. The identification of an interfacial FM layer was attributed to a combination of the effects of strain, Fe deficiency and chemical diffusion from the overlayer. On the other hand, only few experiments on the free surface of FeRh are reported in literature^34^,^35^. In particular, using ultrathin films of FeRh epitaxially grown on a W(100) single crystal, Lee and coworkers^35^ investigated the spin polarization of the valence band of the Fe-terminated surface. Spin polarized angular resolved photoemission experiments concluded that the temperature dependence of the surface magnetic properties is the one expected for the bulk and no evidence of a privileged magnetic state at clean surface was observed. Similarly, surface sensitive photoemission electron microscopy imaging showed homogenous non-magnetic surface of uncapped FeRh films in the AF phase^33^.

The physical properties of a material are strongly dependent on the atomic distribution and relaxation at surfaces and interfaces. In this report, we present a detailed study of the electronic and magnetic properties of a high quality FeRh epitaxial layer, grown on MgO(100), terminated by a plane of Rh atoms. Using synchrotron-radiation spectroscopy techniques with different probing depths we demonstrate the presence of a stable ferromagnetic surface for FeRh at room temperature while the bulk is antiferromagnetic. This experimental finding agrees with first-principles calculations which give a detailed description of the atomic, electronic and spin distribution at the material/vacuum interface.

## Results

### Surface preparation and characterization

**Figure 1:**
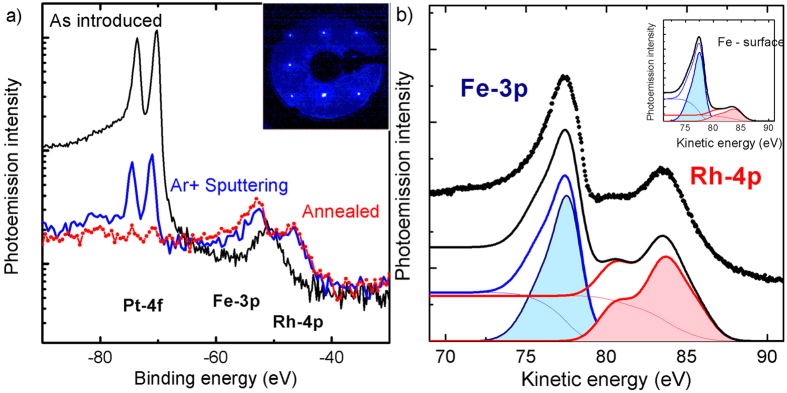
Surface termination (**a**) Photoelectron spectra measured during sample preparation. The black line is the spectrum of the sample as- deposited, the blue line the spectrum after 2 hours of Ar sputtering, and the red dots the spectrum after 30 minutes annealing at 600 K. Inset: LEED pattern measured at 125 eV, after surface annealing. The square lattice of FeRh is clearly visible demonstrating that the surface relaxation preserves the geometry of the atomic planes. (**b**) Fe 3p and the Rh 4p core levels measured on the Rh terminated FeRh system (black dots). The black line is the calculated photoemission intensity using Gaussian line shapes and steps functions (thin red and blue lines) obtained by integration to simulate the corresponding secondary electron background. Filled areas under the red and blue curves are the background subtracted spectral shapes used to calculate the intensity ratio. Inset: Simulation of the photoemission spectrum for a Fe-terminated FeRh surface, to be compared with Fig. 3 of 35.

FeRh(100) films, 50 nm thick, were grown in well controlled way and protected with a 2 nm thick Pt layer grown after cooling down to room temperature. See the Methods section for details. The capping layer was removed by low-energy (500 eV) Ar sputtering in the preparation chamber of the TEMPO beamline at the SOLEIL synchrotron radiation source. The photoemission intensity at Pt 4f was monitored during the removal process using 700 eV photon energy. Black and blue lines in the logarithmic scale of Fig. 1(a) are the photoemission spectra of the as introduced and Ar sputtered sample. The peak at 51 eV binding energy in the as introduced sample is attributed to initial MgO contamination of the protected surface. From cross section and probing depth calculations, the blue curve corresponds to less than 0.5 monolayers of Pt. Annealing at 600 K for 30 minutes leads to Pt diffusion in the layer to reach a non-detectable amount of Pt as indicated by the red curve of Fig. 1(a). A homogenous distribution in the film thickness corresponds to less than 0.1% atomic concentration. The annealed surface shows a square LEED pattern of the FeRh(100) reported in the inset. The procedure was reproduced on several samples, indicating a stable electronic and magnetic properties and low reactivity since no oxidation occurred over several days (the prepared samples were kept in a vacuum better than 5 × 10^10^ mbar).

In the direction normal to the surface, the FeRh film looks like a stacked material composed by alternating planes of Fe or Rh. We determine the atomic arrangement of the sample by comparing the intensities of Fe 3p and Rh 4p photoemission peaks (black dots in Fig. 1(b)). Since these two peaks have similar binding energies (Fe 3p 52 eV and Rh 4p 46 eV), the photoemitted electrons have similar inelastic mean free path λ_IMFP_. When using 130 eV X-ray photons, λ_IMFP_ is close to the minimum, corresponding to 4.5 Å for FeRh^39^.

We use two Gaussian lineshapes with variable spin-orbit splitting, one for each element, to reproduce the spectrum. More complex configurations^36^,^37^ would not be justified for our quantitative analysis. The Fe and Rh components of the fit results are presented as filled color curves in Fig. 1(b). A background step function (thin lines) was obtained by integrating the primary peak for each element. The intensity and the width of the Gaussians were optimized to reproduce the measured photoemission intensity (black line).

The fit procedure allows us to calculate the relative intensity of the Rh and Fe peaks. The ratio between the red and the blue areas in Fig. 1(b) is 0.92. Because of the simple atomic arrangement of the Fe and Rh atoms along the FeRh[100] normal we can model the relative photoemission peak intensity by summing the contributions I_i_ of each atomic plane at position z_i_ weighted by an exponential function with λ_IMFP_ as decay constant:


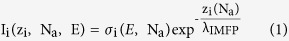


We have used tabulated photoionization cross sections^38^ σ_i_ for each -element N_a_ = (Fe,Rh), photon energy E, and calculated electron inelastic mean free path^39^ λ_IMFP_ . We applied this model considering the two possible ideal terminations of the film. The value obtained for the Rh-terminated surface is 0.88, which is in good agreement with the value extracted from fit parameters of the experimental spectrum in Fig. 1(b). The discrepancy with the value extracted from the measurement indicates a slight Rh abundance. For a Fe-terminated surface the model instead predicts a ratio of 0.21. In the inset of Fig. 1(b) we report the simulated spectrum for a Fe-terminated surface. The result is in good agreement with the measurement on FeRh grown on W(100) single crystal done by Lee and coworkers^35^. This quantitative analysis demonstrates surface is Rh-terminated.

### Photoemission and X-ray absorption

Circularly polarized soft X-rays from the TEMPO beamline^40^ were used to study the magnetic properties of the FeRh surface as a function of temperature by measuring spectroscopic signals characterized by different probing depths. In particular, X-ray magnetic circular dichroism (XMCD)^41^,^42^ experiments at the Fe L_2,3_ and Rh M_2,3_ edges performed detecting the sample photocurrent and magnetic circular dichroism in angular distribution (MCDAD), also proportional to the sample magnetization^43^. Even if XMCD probing depth in iron is known to be particularly short^44^, in iron oxides^45^ and alloys^46^ it is larger than the soft X-ray inelastic mean free path in photoemission. The XPS signal from the Fe 3p core level^43^ measured with excitation energy of 700 eV has a probing depth of about 1.2 nm^39^. In all the experiments the FeRh film is magnetized in the horizontal plane and the circularly polarized photon impinges on the sample with an angle of 42 degree. With this geometry, the electron-energy analyser measures photoelectrons along the surface normal. Because of the constraint induced by photoelectron-spectroscopy experiments, all the measurements are performed at magnetic remanent state, after removing applied magnetic field (400 Oe applied for 300 ms).

**Figure 2:**
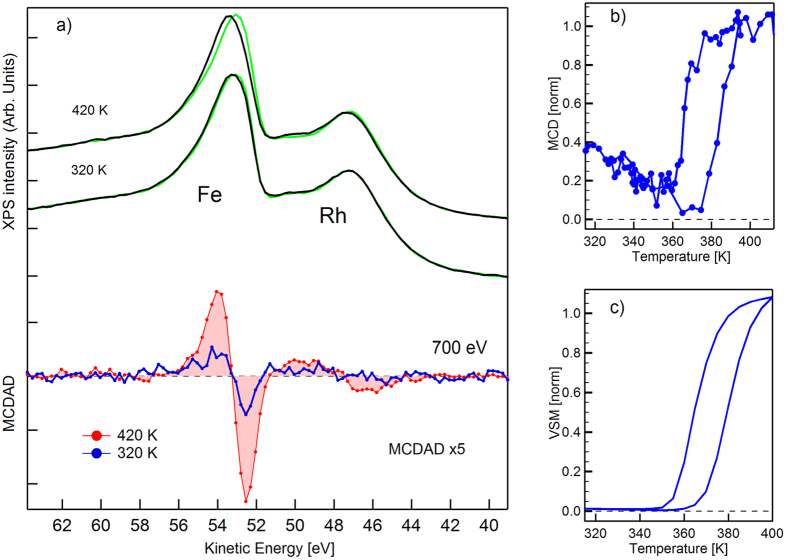
Magnetic Dichroism in photoemission (**a**) FeRh core levels measured at 700 ev excitation energy in the ferromagnetic phase, 420 K and near room temperature, 320 K. The red curve is the MCDAD contrast in the ferromagnetic phase while the blue curve is the MCDAD contrast at room temperature showing the residual magnetism. (**b**) The MCDAD contrast, extracted from the photoemission spectra, plotted as a function of temperature in a heating-cooling cycle. The temperature hysteresis is 5 K larger than the measured for the bulk and slightly shifted toward higher temperatures due to the changes in the interplanar distances induced by surface relaxation. (**c**)Vibrating sample magnetometry (VSM) measured after the sample deposition, showing the temperature dependence of bulk magnetization across the phase transition.

In Fig. 2(a) we report the XPS spectra of Fe 3p and Rh 4p core levels measured at 420 and 320 K with an excitation energy of 700 eV. The black and green curves refer to opposite and parallel directions of the magnetization with respect to photon helicity. The difference curves are reported at the bottom of Fig. 2(a). The red curve is the MCDAD signal extracted from the spectra when the system is in the FM phase. A residual dichroism is observed at 320 K (blue curve), it corresponds to about 25% of the one measured for the FM phase. Similar behavior is observed also for the Rh 4p core level, but the low magnetic signal cannot be used for a quantitative evaluation. The magnetic signal is observed at 320 K, well below the transition temperature. At this temperature the bulk of the system is in the AFM phase. We have measured the temperature dependence of the MCDAD contrast by reversing the magnetization after each scan while slowly cycling the sample temperature between 420 and 300 K, see Fig. 2(b). Each point corresponds to the integral of the positive and negative lobes of the MCDAD curve^36^. The temperature dependence of the FeRh bulk magnetization is well reproduced. The transition temperatures found in XPS are consistent with the Vibrating Sample Magnetometry (VSM) measured after sample deposition, Fig. 2(c). Starting from the FM phase, in the cooling branch a strong reduction of the signal is observed, but the signal recovers back to 25% of the maximum. In the heating branch a reduction of the signal is observed until 380 K where a rapid increase up to the maximum takes place. The observed reduction of the signal is consistent with an increase of the disorder of the moments while approaching the transition temperature suggesting the presence of a surface magnetic layer with a Curie temperature close to 370 K.

**Figure 3:**
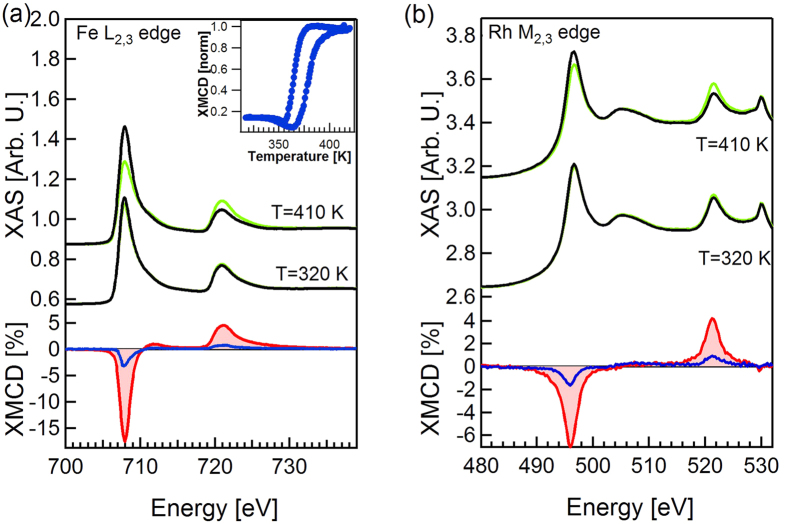
X-ray Magnetic Circular Dichroism (**a**) X-ray absorption (top) and XMCD (bottom) of Fe L_2,3_ edges at 410K, 320K. The dichroism at 320 K (blue curve) is 18% of that measured in the FM phase (red filled curve). (Inset) The XMCD dichroism measured at the Fe L_2_ edge as a function of temperature in a heating-cooling cycle. (**b**) X-ray absorption (top) and XMCD (bottom) of Rh M_2,3_ edges at 410 K, 320 K. For Rh the residual dichroism at 310 K is 23% of that in the FM phase.

To localize the origin of the magnetic signal measured at 320 K analogous experiments were performed using XMCD from the X-ray absorption spectroscopy (XAS) at the Fe L_2,3_ and Rh M_2,3_ edges, which is less surface sensitive. The XAS spectra measured at 410 and 320 K with opposite magnetization directions are reported in Fig. 3(a,b) for Fe and Rh respectively. The XMCD difference signals, in an enlarged scale, are presented below. By applying the XMCD sum rules to the FM phase spectra, and by taking into account the incidence angle of the X rays with respect to the magnetization direction we obtain 2.9 μB for the Fe magnetic moment (we used n_3d_ (Fe) = 6.49 15). It can be compared with the value given by ab initio calculations at 0 K of 3.2 μB. The difference is in good agreement with a 10% reduction expected for a sample with Curie temperature of 675 K^47^. Again a magnetic signal is present at room temperature (320 K, blue XMCD curve) as in the case of photoemission, but now the amplitude relative to the signal measured in the FM phase at high temperature (410 K, red XMCD curve) is 18%. A magnetic signal equal to 23% of the remanent FM value is present also for Rh edge at 320 K, higher than the one observed for Fe. By fixing the photon energy at the maximum of the XMCD signal and reversing the sample magnetization direction with the same procedure used for the photoemission experiment, we measured the temperature dependence of the magnetic signal. The result, which is presented in the inset of Fig. 3(a), reproduces the temperature dependence of the MCDAD contrast in Fig. 2(b). The presence of a residual magnetic moment at room temperature also in Rh demonstrates that there is no formation of ferromagnetic iron domains. In such case we would expect the Rh to be non-magnetic.

## Discussion

**Figure 4:**
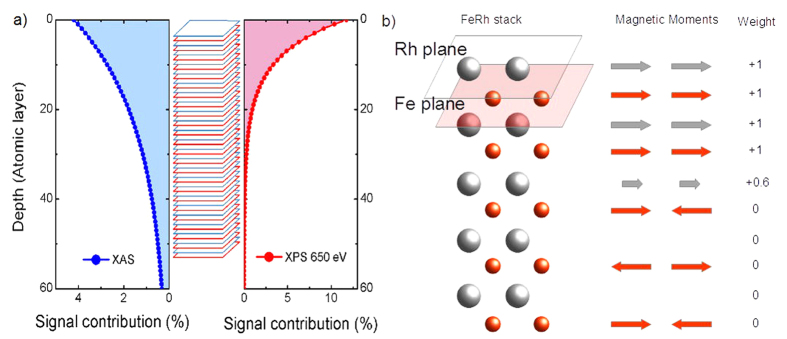
Probing depth comparison. (**a**) Contribution to the total signal intensity of each atomic plane as a function of the plane depth for XAS (blue filled curve) and XPS (red filled curve). (**b**) Left: sketch of a Rh-terminated FeRh stack close to the surface. Center: magnetic moment orientation in the AFM phase with a ferromagnetic layer as simulated in the model. Right: μ_**i**_ values assigned to each plane as a function of its magnetic state.

The measured values of the magnetic dichroism can be fully understood by taking into account the different probing depths of x-ray absorption and photoemission. The contribution of each atomic plane (Fe or Rh) to the total magnetic signal can be calculated by introducing an exponential weight. In analogy with the procedure used to determine the surface termination, we developed a simple model to evaluate the thickness of the magnetic surface-layer taking advantage of the possibility of using two detection techniques with different probing depth. In XPS experiments the probing depth of core-level photoemission can be tuned by changing the photon energy. To optimize the magnetic signal and photon flux we used 700 eV photon energy: the probing depth of the Fe-3p photoelectrons (at 648 eV kinetic energy) is about 1.2 nm^39^. For soft X-ray absorption measured by detecting the total electron yield or the sample current, the probing depth is on the order of 3 nm^48^. The contributions of the atomic planes to the measured XAS and XPS signal are depicted in the left and right panel of Fig. 2(a) respectively. In the center of the Figure the FeRh stack is reported for reference. Since FeRh has a lattice parameter of 2.99 Å the interplane distance is 1.5 Å, hence a 50 nm thick film consists of more than 300 atomic planes. However, only the topmost layers contribute significantly to the signal. For XPS the first two planes contribute to the total signal by almost 22%, while the contribution from deeper planes becomes rapidly negligible. In the case of XAS and XMCD the first plane contributes only for less than the 5%, and a significant contribution to the total signal derives from planes as deep as 60, Fig. 4(a). The contribution of an atomic plane to the magnetic signal is given by the product of a weight times the intensity contribution calculated as in the previous section, Eq. (1):


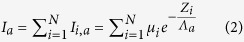


where N is the total number of atomic planes, Λ_a_ and I_a_ are respectively probing depth and intensity of the dichroic signal associated with a specific technique, μ_i_ is the coefficient representing the magnetic contribution, and I_i,a_ is the contribution of a single plane. We assign a weight μ_i_ to each atomic plane, assuming that atoms in the third Rh layer carry only 60% of the expected moment, Fig. 4(b). This assumption will be justified by the results of theoretical calculations reported in the next section. For ferromagnetic Fe and Rh plane μ_i_ is set to 1, while for the antiferromagnetic Fe planes and for the non-magnetic Rh planes μ_i_ is 0. A pictorial representation of the model and of the simplest assignment of the weight values accounting for the observed magnetic signal at room temperature is given in Fig. 4(b). Fe and Rh intensities are calculated separately and in each case the summation index i runs over the positions occupied by Fe and Rh, respectively.

We calculate the intensity of the maximum magnetic signal in the FM phase separately for Fe and Rh assuming that each plane carries the maximum magnetic moment and we use this value to evaluate the percent of signal obtained at 330 K. Using the XPS probing depth we calculate a magnetic signal equal to 35% of the one observed in the ferromagnetic phase. This can be compared with the 31% extracted from the experiment. For the XAS case, the model gives a residual moment of 17.6% for Fe and 22.1% for Rh, to be compared with the experimental values of 18% and 23%, respectively. The agreement of both XPS and XMCD signals with the results of the model is very good with only a 10% of uncertainty. Using a decay length of 1.2 nm for the photoemission and 3.4 nm for x-ray absorption^48^, the measured magnetic signals can be reproduced assuming an antiferromagnetic bulk and a surface ferromagnetic phase composed of the first 2 Fe and 3 Rh atomic planes.

### Theoretical description

To gain a microscopic confirmation of this experimental indication, we performed first-principle calculations within density functional theory (DFT) in the local-density approximation (LDA)^49^. By fully taking into account the electronic and interatomic interactions, DFT allows one to determine, in principle in an exact manner, the electronic ground state and the equilibrium crystal structure at T = 0 K of real materials. In practice, previous studies^11^,^25^,^26^,^50^,^51^,^52^,^53^ have assessed the LDA to be a reliable approximation for FeRh.

**Figure 5:**
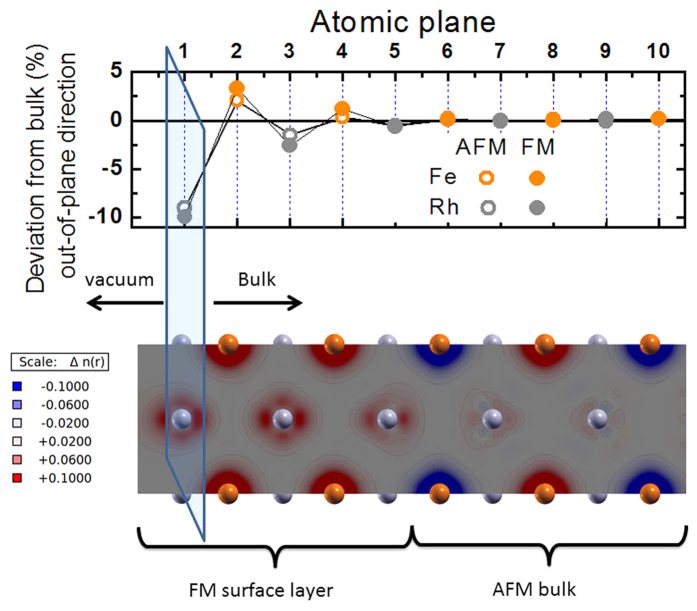
DFT-LDA theory. (Top) Calculated deviations of the out-of-plane interatomic distances from the bulk values. Open and full circles refer respectively to the AFM and FM phase. The positions of the 5 topmost atomic planes are significantly modified: Rh planes undergo a compressive displacement (negative shift) while for Fe planes is expansive (positive shift). (Bottom) Spin density distribution of a relaxed FeRh slab. Grey and red spheres identify Rh and Fe atoms, aligned with the top panel, respectively. The FM surface layer involves the first 5 atomic plane while deeper planes behave as in the bulk, remaining in the AFM phase.

We simulated the FeRh(100) surface by considering a slab consisting of 16 alternating atomic planes of Fe and Rh in the FM and type-II AFM configurations^50^. In the latter, Fe atoms are aligned antiferromagnetically within each layer containing two inequivalent atoms per unit cell. The top panel of Fig. 5 shows the displacements in the out-of-plane direction of Fe and Rh atoms in the slab model (represented as red and grey circles respectively), as an effect of the structural relaxation from bulk positions (only the topmost 10 planes starting from the Rh surface are shown for better clarity). We find that while the central planes remain equidistant as in the bulk, the surface planes undergo a sizeable displacement, confirming that the surface properties in FeRh can be expected to differ from the bulk. In particular most external Rh planes move toward the bulk (the deviations from bulk position are negative, top panel of Fig. 5) while the Fe planes move toward the vacuum (the deviations from the bulk are positive). This occurs independently of the magnetic configuration, for both the AFM and FM qualitatively similar displacements are observed. As a result of this structural relaxation, while surface interplanar Rh-Rh distances decrease, Fe-Fe distances increase. Since also in the bulk Fe-Fe distances are greater in the FM phase than in the AFM phase, this result suggests that from the structural point of view the surface layers relax towards the bulk FM state.

In the relaxed structures, besides the FM and AFM configurations, we also simulated a mixed magnetic configuration in which the 2 topmost Fe layers in the Rh terminated surface are ferromagnetic and the remaining 6 are antiferromagnetic. The different self-consistent solutions at 0 K are found to be within 9 meV/atom energy, suggesting a phase space with several local minima separated by shallow barriers. Moreover, in all simulations the initial magnetization for Rh atoms was always set different from zero. In the fully AFM configuration, both in the bulk and in the slab, Rh local moments at self-consistency always converge to zero. This is due to a magnetic frustration induced by hybridization with Fe atoms with opposite moments located at equal distances from Rh atoms^26^,^52^. On the contrary, in the slab with the mixed magnetic configuration, Rh atoms in the surface layers still preserve a finite magnetic moment, as shown in the bottom panel of Fig. 5, where the magnetization density is depicted (red and blue contours represent positive and negative values, respectively). We find that in the 2 topmost surface Rh layers (grey balls) the magnetic moments (1.0 μ_B_ each, as in the bulk FM phase) are ferromagnetically aligned. Moreover, also in the third Rh layer, which is sandwiched between a FM and an AFM Fe layer (red balls), a finite magnetic moment of 0.6 μ_B_ still survives. In the other Rh layers, the magnetic moments go to zero (even though the magnetization density around Rh atoms is not zero, as discussed in^26^). Therefore, the results of the calculations are consistent with the existence of the FM surface layer carrying the magnetic moments of the bulk FM phase, which has been experimentally discovered.

**Figure 6:**
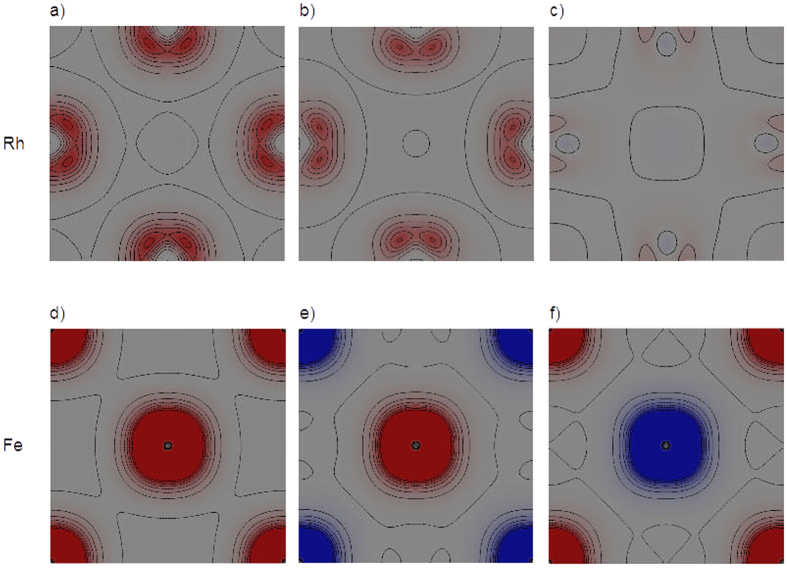
Site Specific Magnetization Maps. Calculated magnetization density (depicted as red/blue contours) for Rh planes panels (**a–c**) and Fe planes panels (**d–f**).We selected the position of the sections to show the most representative behaviour of the magnetization in the different atomic planes.

Figure 6 displays different sections parallel to the FeRh(100) surface of the magnetization density distribution. In particular, each section corresponds to a different atomic plane of FeRh that is stacked as shown in Fig. 5. The magnetization density is always distributed around the atoms (not shown explicitly in the picture for sake of clarity). The top line of the figure shows three representative planes containing Rh atoms (a–c), while the bottom line shows Fe planes(d–f). Concerning the Rh planes (top line), panel (a) shows the magnetization in the first two ferromagnetic Rh surface planes. Panel (b) is the Rh plane that is sandwiched between a ferromagnetic and an antiferromagnetic Fe plane, where Rh atoms have a magnetic moment that is 60% of the surface ferromagnetic Rh plane. Panel (c) is the plane where the Rh atoms have an average magnetic moment equal to zero. It shows that also in this case the magnetization is not strictly zero everywhere as discussed in^26^. Concerning Fe planes (bottom line), panel (d) is representative of the first two surface ferromagnetic Fe planes, whereas the other two panels (e and f) show the alternating antiferromagnetic Fe planes in the bulk, according to the type-II antiferromagnetic configuration of FeRh.

## Conclusions

In conclusion, we have given experimental evidence that the Rh-terminated surface of FeRh is ferromagnetic at room temperature while the bulk is in the AFM phase. A simple phenomenological model based on the assumption of different probing depths for XAS and photoemission indicates that 5 atomic planes, 3 of Rh and 2 of Fe, are ferromagnetic. First-principle calculations provide a microscopic description of the surface structural relaxation and of the spin-density distribution indicating the stability of the 5 FM planes at the Rh-terminated surface, thus supporting the interpretation of the experimental data.

These results show how surface and interface relaxation can induce specific electronic and magnetic configurations and explain how several results attributed to chemical bonding with the capping layer can be also related to the relaxation process at the interface. Furthermore the findings suggest that it is possible to tailor a well-confined magnetic thin layer by modifying the atomic arrangement of the sample. A study of the modifications induced by the atomic interaction with other materials is necessary for a complete understanding of the interface magnetic behavior.

## Methods

FeRh(100) films, 50 nm thick, were grown epitaxially on MgO(100) substrates by dc magnetron sputtering using an equiatomic target. The films were grown at 725 K and post-annealed at 1070 K for 45 minutes. Temperature-dependent vibrating sample magnetometry (VSM)(see Fig. 2(c)) shows the expected hysteretic behavior of the sample magnetization suggesting a homogeneous and ordered B2 phase^23^,^54^,^55^.

Spectroscopic investigations were performed on the UHV photoemission experimental station of the TEMPO beamline at the SOLEIL synchrotron radiation source. Two Apple II insertion devices deliver circularly polarized radiation at low (HU80) and high (HU44) photon energy which is selected using a high-resolution plane grating monochromator, with a resolving power E/ΔE that can reach 15000 on the whole energy range (45–1500 eV). During the XPS measurements, the photoelectrons were detected at 0° from the sample surface normal 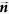 and at 45° from light propagation direction. Sample was magnetized in the surface plane with a horseshoe electromagnet. The soft x-rays spot size was 80 × 40 (H×V) μm.

In the DFT calculations we adopted a supercell approach (10 Å vacuum was enough to prevent interactions between different replicas) using the Abinit plane-wave code^56^,^57^. We employed norm-conserving Hartwigsen-Goedecker-Hutter (HGH) pseudopotentials^58^. Calculations were converged with a 65 Hartree cutoff (115 Hartree when semicore Fe 3s and 3p and Rh 4s and 4p were explicitly considered as valence electrons) and a 8 × 8 × 1 k-point grid. All atomic positions were relaxed until interatomic forces were less than 2.5 × 10^−3^ eV/A. Results for the bulk are in line with previous results from literature^11^,^24^,^25^,^26^,^50^,^51^,^52^ and in good agreement with experiment. At 0 K the AFM phase is more stable by 36 meV/atom than the FM phase and the calculated lattice parameters are underestimated by 1% with respect to experimental data^14^. The magnetic moments obtained from the integration of the magnetization density over a sphere of 2.1 a.u radius for Fe and 2.4 a.u. for Rh are 3.2 μ_B_ for Fe and 1.0 μ_B_ for Rh in the FM phase and become respectively 3.1 μ_B_ and 0 μ_B_ in the AFM phase.

## Additional Information

**How to cite this article**: Pressacco, F. *et al.* Stable room-temperature ferromagnetic phase at the FeRh(100) surface. *Sci. Rep.* **6**, 22383; doi: 10.1038/srep22383 (2016).
